# CNS Demyelination with TNF-α Blockers

**DOI:** 10.1007/s11910-017-0742-1

**Published:** 2017-03-21

**Authors:** Elissavet Kemanetzoglou, Elisabeth Andreadou

**Affiliations:** 1 Department of Neurology, Agii Anargiri General Oncological Hospital of Kifissia, Athens, Greece; 2 1st Department of Neurology, Athens National and Kapodistrian University, Aeginition Hospital, 74, Vas. Sophia’s Ave, Athens, Greece

**Keywords:** Anti-TNF-α, Demyelination, Tumor necrosis factor, Multiple sclerosis

## Abstract

Tumor necrosis factor–α (TNF-α) blockers are a popular therapeutic choice in a number of inflammatory diseases. Thus far, five TNF- α blockers have been approved for clinical use (etanercept, infliximab, adalimumab, golimumab. and certolizumab). Despite being considered relatively safe, serious side effects associated with immune suppression have been reported, including central and peripheral nervous system (CNS) demyelinating disorders. It is still elusive whether these events are mere coincidence or a side effect of anti-TNF-α use. In this paper, we review the published case reports of CNS demyelination associated with anti-TNF-α therapy and present the follow-up of our 4 previously reported patients who developed neurologic symptoms suggestive of CNS demyelination after having received anti-TNF-α treatment. We also discuss the possible role of TNF-α blockers in demyelination.

## Introduction

Tumor necrosis factor-α (TNF-α) blockers present a revolutionizing therapeutic choice for a number of inflammatory diseases such as Crohn’s disease (CD), ankylosing spondylitis (AS), and rheumatoid arthritis (RA). Several terms such as TNF-α blockers, anti-TNF-α agents, TNF-α antagonists, and TNF-α inhibitors are used interchangeably, all describing the same biological agents. Henceforth in our paper, we will use the term TNF-α blocker for clarity.

These agents are more effective than traditional disease modifying drugs (DMDs), controlling disease activity and preventing underlying structural tissue damage. Although they are relatively safe, an increasing number of neurologic side effects have been reported in the literature, consisting of central and peripheral nervous system demyelinating events. These adverse events suggest a possible relationship between anti-TNF-α use and demyelination [1]. However, it remains uncertain whether these episodes are coincidental or causally linked, or how TNF-α blockage may possibly trigger or exacerbate demyelination.

In this paper, we review the pathogenic and protective functions of TNF-α, the role of TNF-α blockers in CNS demyelination and the CNS demyelinating cases reported in the literature to be related to anti-TNF-α therapy. We also present the follow-up of our 4 previously reported patients [2] who developed neurologic symptoms suggestive of CNS demyelination after having received TNF-α blockers.

## TNF-α Mechanism of Action

TNF-α is a pleiotropic cytokine, with a wide range of functions: homeostatic, immune, and inflammatory. The beneficial homeostatic functions of TNF-α include defense against pathogens, development of lymphoid organ architecture, resolution of inflammation, tissue regeneration, immune regulation, and inhibition of tumor growth. The pathogenic functions of TNF-α comprise triggering of inflammation, stimulation of vascular endothelium, proliferation of immune cells, and tissue damage [3, 4]. Under physiological conditions, macrophages, lymphocytes (T and B), natural killer cells, dendritic cells, and monocytes produce TNF-α in the periphery [5], while in the CNS TNF-α is produced mainly by migroglia, neurons, and astrocytes [4–7].

TNF-α is produced initially as a transmembrane molecule (tmTNF). Subsequently, it is released from cells as a soluble cytokine (sTNF) via regulated cleavage of tmTNF by TNF-α converting enzyme (TACE). Both forms of TNF-α are biologically active and interact with two receptors (TNFR1 and TNFR2) with different affinity. sTNF has a higher affinity for TNFR1, contains a death domain, and mediates apoptosis and chronic inflammation [8]. tmTNF has a higher affinity for TNFR2, activating genes important for cell survival, resolution of inflammation, and even myelination. TNFR1 is expressed in all cell types, whereas TNFR2 is expressed mainly on neurons, immune cells, and endothelial cells [9••, 10, 11].

At low levels in tissues, TNF-α exerts beneficial homeostatic functions, as it enhances host defense mechanisms against intracellular pathogens, particularly mycobacteria [12]. At elevated concentrations, TNF-α can promote inflammation and organ injury [13]. In disease states, TNF-α is considered to be a proinflammatory cytokine that is promptly produced in response to stimuli, both systemically and locally in the affected tissues, predominantly by activated macrophages and monocytes [13]. Moreover, circulating TNF-α crosses the blood brain barrier (BBB) into the brain [14]. Inflammatory stimuli in the CNS induce TNF-α production mainly by microglia, neurons, and infiltrating immune cells [9••].

### Types and Mechanism of Action of TNF-α Blockers

Five anti-TNF-α blockers are approved for clinical use: etanercept (circulating receptor fusion protein), infliximab, adalimumab, and golimumab (IgG monoclonal antibodies), and certolizumab (PEGylated Fab1 fragment of an IgG1 monoclonal antibody) [15]. Both receptor and antibody based TNF-α blockers act as antagonists by blocking tmTNF interactions with TNFR1/2, and as agonists, by inverting signal leading to apoptosis, cell activation, or cytokine inhibition [5, 16, 17]. Etanercept also binds to lymphotoxin LTα3, which is structurally similar to sTNF, with equivalent or greater affinity than sTNF [16].

Compared with the traditional DMDs, TNF-α blockers are more efficacious, with faster onset of action and more effective control of disease progression. These characteristics have made them an appealing option in refractory cases [18, 19].

### Clinical Uses of TNF-α Blockers

TNF-α blockers present a revolutionizing therapeutic choice for inflammatory diseases such as RA, AS, plaque psoriasis, psoriatic arthritis, juvenile polyarticular rheumatoid arthritis, and inflammatory bowel disease (CD and ulcerative colitis). TNF-α blockers are also used off-label for a number of other inflammatory conditions such as sarcoidosis, hidradenitis suppuritiva, Adamantiades-Behcet's disease, pyoderma gangrenosum, dermatomyositis, scleroderma, noninfectious uveitis, and others [20, 21]. Novel indications for the use of TNF-α blockers are under investigation, and new TNF-α blockers are being evaluated.

### Anti-TNF-α Blockers’ Trials for Multiple Sclerosis

Initial studies in experimental autoimmune encephalomyelitis (EAE) animal models of multiple sclerosis (MS) showed beneficial effects of TNF-α blockers. Subsequently, given their anti-inflammatory effects, clinical trials of infliximab [22] and lenercept [23] (a receptor-based TNF-α blocker) were carried out in people with MS with surprisingly unfavorable results. Specifically, in an open label phase I trial with infliximab, 2 patients with rapidly progressive MS showed increased disease activity and MRI lesion load [22]. Furthermore, a randomized double-blind placebo-controlled multicenter trial in 168 relapsing-remitting MS patients with lenercept was stopped prematurely due to a dose-dependent increase in relapse rate, attack duration, and severity of exacerbations [23].

These trials suggested that non-selective inhibition of TNF-α is harmful in MS and that TNF-α exerts both potent pro-inflammatory effects and essential protective functions in the CNS under pathological conditions [9••].

### Side Effects of TNF-α Blockers

The efficacy of TNF-α blockers in inflammatory conditions has increased their use. Despite their relatively safe profile, well-known adverse events include injection site reactions, risk of infections (especially tuberculosis reactivation), congestive heart failure, hemocytopenia, and T-cell lymphomas [24, 25]. Reports of autoimmune diseases, including lupus-like syndromes and vasculitis [26], diabetes mellitus, psoriasis, interstitial lung diseases, sarcoidosis, autoimmune hepatitis, uveitis, antiphospholipid syndrome, myositis, and myasthenia gravis have also been published [1, 27–29].

With the widespread use of TNF-α blockers, a growing number of demyelinating events have been reported, including CNS demyelinating disorders [MS, optic neuritis (ON), acute transverse myelitis (TM)], as well as peripheral nervous system disorders (Guillain-Barré syndrome, Miller Fisher syndrome, chronic inflammatory demyelinating polyneuropathy, multifocal motor neuropathy with conduction block, mononeuropathy multiplex, and axonal sensorimotor polyneuropathies) [1, 30]. It still remains unclear whether these events are coincidental or are actually side effects of anti-TNF-α use. Even more elusive remains the underlying pathogenic mechanism [3].

### TNF-α Effect on Demyelination

The pleiotropic functions of TNF-α often show contradictory effects, particularly in the CNS. TNF-α and its receptors can either promote neuroinflammation and secondary neuronal damage, or exert protective functions under pathological conditions. Furthermore, TNF-α exerts distinctive actions at different stages of autoimmune demyelination: sTNF, but not tmTNF, promotes inflammation and disease onset, whereas sTNF and/or tmTNF have protective functions in established disease by reducing the extent and severity of autoimmune inflammation. sTNF mediates the proinflammatory effects of TNF-α via TNFR1 signaling. TNFR1 plays a critical role for the onset of CNS autoimmune disease, through induction of a pro-inflammatory environment in the CNS, but subsequently suppresses local inflammation either indirectly, mediating neuroprotection, or directly, promoting repair processes [9••]. Elevated production of TNF-α was observed in patients and animal models of MS. TNF-α overexpressing transgenic mice develop spontaneous demyelination that reverses with anti-TNF-α administration [9••], whereas demyelination is delayed in TNF-α deficient mice [31]. TNF-α has been found to increase permeability of the BBB [14] and high levels of TNF-α were found in active central and peripheral demyelinating lesions [32, 33] as well as in the serum, cerebrospinal fluid (CSF), and brain plaques of MS patients [34] correlating with disease severity or exacerbation [35, 36]. It was recently shown that TNF-α is predominantly produced by both macrophages and microglia during acute EAE and is decreased during remission, whereas TNF-α is sustained by infiltrating macrophages in progressive EAE, enhancing clinical disability and CNS inflammation [37•]. TNF-α has also been implicated in promoting macrophage polarization to a pro-inflammatory M1 phenotype [38•]. Additionally, TNF-α seems to exert a protective role in the periphery and a pathogenic role in the CNS by suppressing encephalitogenic T cell production of Th1 and Th17 in lymphoid organs, while promoting immunocyte infiltration in the CNS through chemokine production, exacerbating disease severity [39]. Inhibition of both sTNF and tmTNF does not seem to protect against demyelination [8].

Besides being crucial in host defense and inflammation and accelerating acute demyelinating processes, TNF-α is also necessary for triggering remyelination [8, 39] and promoting the proliferation of oligodendrocyte precursor cells [40]. In later stages of MS, TNF-α has shown immunosuppressive properties. In early studies of animal models, inhibition of TNF-α resulted in EAE improvement or protection against demyelination [31, 41–43]. In recent studies, selective inhibition of sTNF was found to be beneficial during EAE, implying that the protective effects of TNF-α are exerted through the interaction of tmTNF with TNFR2 [8]. Evidence from EAE models suggest that remyelination in the CNS necessitates the expression and activity of TNFR2 and CXCR4 by oligodendrocyte progenitor cells, promoting their proliferation and differentiation into mature oligodendrocytes [40], and that selective inhibition of tmTNF/TNFR2 leads to demyelination and oligodendrocyte apoptosis [44].

Interestingly, MS susceptibility has been associated with a single nucleotide polymorphism (SNP) in a gene, encoding TNF receptor 1 (TNFR1), the *TNFRSF1A* gene. This SNP in TNFR1 leads to expression of a soluble form of TNFR1 that inhibits TNF-α in humans and this may play a role in MS development in some individuals, possibly mimicking the effects of TNF-α blockers [45].

### Theories About the Mechanism of Action of Anti-TNF-α Therapy in CNS Demyelination

Since TNF-α is implicated in demyelinating processes, TNF-α blockers were considered as a potential therapeutic choice in MS. However, the negative outcomes of these agents in MS trials [22, 23] and the reports of demyelinating events following their use for other disorders raised the suspicion that use of these drugs could be a risk factor of demyelination. In an attempt to clarify the potential biological role of TNF-α blockers in triggering or aggravating demyelination, several theories have been proposed [46]:

1. TNF-α blockers cannot penetrate the intact BBB to suppress demyelination but they can enhance demyelination through increased ingress of peripheral autoreactive T-cells into the CNS (lack of entry theory). This theory provides a possible explanation for the failure of anti-TNF-α blockers in reducing demyelination and for their effect on aggravating MS [3, 47].
2. TNF-α blockers may aggravate CNS demyelination by decreasing TNFR2 receptors, which are necessary for the proliferation of immature oligodendrocytes and myelin repair [48–54].
3. TNF-α blockers could alter cytokine responses by downregulating interleukin-10 and upregulating interleukin-12 and interferon-γ, creating a profile similar to that of MS patients [54, 55•, 56].
4. TNF-α blockers may deactivate TNF-α systemically, but not within the CNS (due to BBB impermeability), leading to a high concentration of TNF-α in the CNS (“sponge effect”) [3, 47].
5. There may be systematic dysregulation of TNF-α in patients with RRMS, as was shown in a recent study of Mausner-Fainberg et al [57••], in which increased serum neutralization capacity of TNF-α in RRMS patients was observed. These findings offer a possible explanation for the demyelinating events after TNF-α blockade.
6. Finally, TNF-α blockers may unmask an underlying latent infection, which can lead to autoimmune demyelination [5, 47, 58, 59].

## Reviewed Cases

We present the follow-up of our 4 previously reported patients, 2 with RA and 2 with AS who developed neurologic symptoms following anti-TNF-α exposure [2]. We also review the cases of CNS demyelination associated with TNF-α blockers, published in the medical literature between January 1990 and August 2016. We conducted a PubMed literature search of available material on documented CNS demyelination in patients receiving TNF-α blockers. Articles from PubMed were obtained using the search terms “Demyelinating Disease,” “Multiple Sclerosis,” “Optic neuritis,” and “Tumor Necrosis Factor-α.” Including our 4 patients, 122 cases with CNS demyelinating events during anti-TNF-α treatment were identified: 69 case reports and 53 cases from the Spanish Registry of biological therapies in rheumatic diseases and an adverse event data base [30, 49, 50, 56, 60–90, 91•, 92••, 93].

All cases are summarized in Table 1; 75 patients were female (61%) and 47 were male (39%) with a mean age of 45.29 years (SD: 14.89). Only 3 patients were reported to have a family history of MS; 61 patients (50%) had RA, 14 (11%) had AS, 20 (16%) had PsA, 10 (8%) had CD, and 18 (15%) had other rheumatologic and inflammatory diseases. Fifty (41%) patients were treated with infliximab, 57 (47%) with etanercept, 19 (16%) with adalimumab, and 1 (1%) with golimumab. Three patients (2.5%) received more than one TNF-α blocker successively. Seven patients (6%) were receiving combined therapy with TNF-α blockers and methotrexate (MTX) at the onset of symptoms; 28 patients (23%) had received different DMDs before the initiation of anti-TNF-α treatment, mostly MTX. The mean time of exposure to TNF-α blockers, before the onset of symptoms, was 17.61 months (SD: 18.07, range: 3 d–6 y). According to previous studies, the interval between anti-TNF-α initiation and onset of symptoms was approximately 5 months (1 wk–15 mo) [30]. At presentation, diagnosis of MS was confirmed in 26 patients (21%), ON in 46 (38%), monophasic demyelinating event (MDE) in 37 (30%), progressive multifocal leukoencephalopathy (PML) in 3 (3%), tumefactive lesions in 2 (2%), TM in 6 (5%), and leukoencephalopathy in 2 (2%); 39% of the cases were treated with pulse steroid therapy (48 patients), whereas oral steroids were administered to 12% of patients (15 patients). The mean follow-up time was 12.78 months (SD 12.71). Interestingly, 7 patients were diagnosed with MS after longer follow-up (mean follow up 20.43 months). TNF-α blockers were discontinued in all except 2 patients, where available information is missing. In 1 patient treatment was restarted with a positive rechallenge phenomenon. Complete recovery after the initial therapy was reported in 44 patients (36%) and partial in 26 patients (21%), whereas no resolution of symptoms was described in 34 patients (28%). Two patients with PML (1.6%) and 1 with MDE (0.8%) died.

**Table 1:** Demographical, clinical, and imaging data of the reviewed cases

CASE REPORTS														
citation	Sex/age	Family history of MS	diagnosis	Previous treatments	antiTNFa	Exposure time (m)	Type of demyelination	MRI lesion	OCB	Anti-TNF-a cessation	Therapy	Recovery	Disease progression	Total followup (m)
50	F/33	No	RA	NR	ETN	24	MS	Brain, CSC gd +	NR	Yes	none	Partial (4 m)	MRI progression (+gd)	12
51	F/47	Yes	PsA	MTX, LEFL, folic acid	IFX	7	MS	Brain, SC gd+	Yes	Yes	Pulse MP	partial	After 1y new relapse steroids = IVIG no improvement. Therapy MITO	12
57	F/40	No	RA	MTX	ADM	2	MDE	Brain + SC gd+	Yes	Yes	Pulse steroids	Complete	No	14
62	F/45	NR	CD	NR	IFX	10	ON	NR	NR	Yes	Pulse steroids	Complete (3 m)	No	3
62	F/55	NR	RA	NR	IFX	13	ON	NR	NR	Yes	Pulse steroids	Complete (3w)	no	0,75
62	M/54	NR	RA	NR	IFX	3	ON bilateral	NR	NR	Yes	Pulse steroids	No	NR	NR
62	F/62	NR	RA	NR	IFX	3	ON bilateral	NR	NR	Yes	Pulse steroids	No	NR	NR
62	M/54	NR	RA	NR	IFX	2	ON bilateral	NR	NR	Yes	Pulse steroids	No	NR	NR
62	F/50	NR	CD	NR	IFX	NR	ON	NR	NR	Yes	None	Complete (1,5 m)	no	1,5
62	F/45	NR	RA	NR	IFX	11	ON	NR	NR	Yes	Pulse steroids	Complete (3 m)	no	3
62	F/12	NR	JIA	NR	ETN	2,5	ON	NR	NR	Yes	Pulse steroids	Complete (3 m)	no	18
62	F/17	NR	JIA	NR	ETN	8	ON	NR	NR	Yes	Pulse steroids	Complete (2 m)	no	20
62	F/21	NR	JIA	NR	ETN	18	ON bilateral	NR	NR	No – ETA	Pulse steroids	No	no	6
62	M/18	NR	JSpA	NR	ETN	11	ON	NR	NR	Yes	Pulse steroids	Complete (1w)	no	14
62	F/31	NR	RA	NR	IFX	4	ON	NR	NR	Yes	Pulse steroids	Complete (1 m)	no	12
62	M/55	NR	RA	NR	ETN	3	ON bilateral	Brain gd-	NR	Yes	Pulse steroids	Complete (1w)	no	12
62	M/55	NR	PsA	NR	ADM	4	ON	NR	NR	Yes	Pulse steroids	Complete (1w)	no	12
62	M/40	NR	RA	NR	ADM	12	ON	Brain + SC gd-	NR	No –ADA	None	Partial		4
63	M/48	NR	CD	MTX, SUL, LEFL, PREDN	ADM	12	ON	Brain ON	NR		Oral steroids	Complete (1 m)	No	12
64	F/48	NR	RA	NR	ETN	42	TM + PM	Brain nm	No	4 m after	Oral steroids + amitriptyline	No	No	6
65	M/66	NR	RA	LEFL, HCQ, MTX	ADM, MTX	3	leukoencephalopathy	Brain gd-	No	2 m after	Oral steroids + meloxicam	Partial	No	1,67
66	F/66	NR	RA	DMARDs	ETN	24	MDE	Brain + CSC gd+	No	Yes	Pulse steroids	Complete	No	1
67	F/32	NR	RA	MTX	ADM	23	ON	NR	NR	Yes	Pulse steroids	Partial	MRI progression After 3 m gd+, MS diagnosis	3
68	F/68	No	RA	GST, D-P, Buc, Sal MTX, PREDN, NSAIDs	ADM	23	MS	Brain	NR	3w prior	None	Complete recovery	2w after MRI + gd	12
69	M/56	NR	RA	PREDN, MTX, Buc	IFX, MTX, PREDN	2,5	MDE	Brain, CSC gd +	No	Yes	Pulse steroids	Complete	no	9,5
69	F/66	No	RA	PREDN, MTX	IFX, MTX, PREDN	4	MDE	Brain, SC gd+	No	Yes	Oral PREDN	Partial	no	3
70	F/58	NR	RA	MTX	ETN	>12	MDE	CSC gd-	Yes	Yes	Pulse steroids	Complete 3w	no	12
71	F/41 (ON 9y before)	NR	AS	Indomethacin, PREDN	ETN	6	MS	Brain gd-	Yes	Yes	none	Complete	no	3
72	F/5	NR	Still	Naproxen, MTX, steroids	ETN	12	MDE	Brain gd +	No	Yes	NR	NR	NR	6
73	M/43	No	RA	NR	IFX	24	MDE	Brain gd -	No	Yes	None	Complete 1 m	RA worsened	16
73	F/47	No	RA	PREDN, MTX	ADM	48	MDE	Brain gd-	Yes	5 m later	None	No	No	2
73	F/49	No	RA	MTX	ETN	11	MDE	Brain gd+	Yes	1 m later	none	No	RA progression (MTX, HCQ). New MS relapses (INFb1a) MS diagnosis	18
74	F/42	NR	CIAU	MTX, mycophenolate mofetil	ADM	0,75	MS	Brain gd+	Yes	Yes	Pulse MP	Complete (<1 m)	immunomodulatory therapy	6
75	F/53	No	RA	PREDN, MTX, SULF, LEFL	IFX	1,5	ON	NR	NR	NR	Pulse steroids	Complete	NR	NR
76	M/53	No	PsA	PREDN, MTX, NSAIDs	ETN,MTX	6	MDE	Brain, SC gd-	Yes	Yes	INFb 3times/w + baclofen	Partial	Symptom progression., MS diagnosis Steroids and INFb1a	24
76	M/42	No	PsA	MTX, steroids, SULF	ETN	21	MDE	CSC gd+	No	Yes	MTX + daily steroids		RA poorly controlled, symptom progression MS diagnosis	36
76	F/51	NR	AS HLAB27+	MTX, PREDN, NSAIDs	ETN	18	MS	Brain, SC gd+	No	Yes	None	partial	No	24
77	F/36	No	PsA	MTX, PREDN	ETN	4	ON	Brain gd-	No	Yes	Pulse MP	Partial 1w	After 6 m peripheral facial palsy and MRI progression	6,5
78	F/35	Yes	UC	SULF, PREDN, 6-MP, CYC	IFX	18	MS	Brain, SC gd +	Yes	Yes	None	NR	NR	NR
79	F/32	NR	JRA	NR	ETN	2	Autoimmune leukencephalopathy	Brain gd-	Yes	Yes	Pulse steroids, PLEX, IVIG, CYC, AZA, PREDN	partial	No	12
79	F/51	NR	CD	NR	IFX, ADM	IFX 12, ADM 24	TM	Brain gd-	NR	Yes	None	No	No	10
79	F/61	NR	RA	NR	ETN	48	ON	Brain gd +	NR	Yes	None	Partial	No	6
79	F/42	NR	PsA	NR	ETN, ADM	ETN 48, ADM 4	ON bilateral	Brain, SC gd+	NR	Yes	Pulse steroids	partial	No	8
79	F/27	NR	CD	NR	IFX, ADM	IFX 36, ADM 6	Polycranial neuritis, rhombencephalitis,	Brain gd+	Yes	Yes	None	Partial	No	6
79	F/61	NR	NLD	NR	IFX	9	TM	Brain,SC gd+	No	Yes	Pulse steroids	Partial	No	6
79	F/61	NR	Ps	NR	ETN	72	TM	Brain, SC, gd -	NR	Yes	Glatiramir acetate, INFb-1a	No	Progressive symptoms, MS diagnosis	36
79	F/44	NR	AS, Graves	NR	IFX	7	TM	SC gd-	-	Yes	Pulse steroids	partial	No	18
79	F/69	NR	RA	NR	IFX	7	TM	SC gd-	-	Yes	Pulse steroids	partial	No	18
80	M/55	NR	PsA	MP	ETN	26	MDE	Brain, SC gd -	+	Yes	Pulse steroids	No	LEFL	3
80	M/44	NR	AS HLAb27+, Ps, UC	NR	ADM	16	ON bilateral	Brain gd-	-	1 month later	None	NR	NR	NR
81	F/26	No	CD	6-MP, steroids	IFX ADM	IFX 6 ADM 4	MS	Brain gd -	Yes	Yes	Pulse steroids	No	New MRI lesions at 6w = PLEX	48
82	M/57	NR	RA	NR	IFX, MTX, PREDN	4	MDE	Brain gd -	No	Yes	Pulse MP, PLEX	DIED	-	-
83	M/69	NR	RA	CYC, HCQ, steroids,	IFX	NR	PML	Brain gd -	NR	Yes	None	DIED	-	-
84	M/72	NR	RA	PREDN, HCQ, MTX	IFX, MTX, PREDN	60	PML	Brain gd-	NR JC -	2,5 m later	Supportive therapy	No	No	NR
85	F/23	NR	SLE, RA	PREDN, HXQ	ETN	48	PML	Brain gd -	NR JC+	Yes	Cytosine arabinoside	DIED	-	-
86	M/44	NR	AS HLAB27+	MTX, LEFL	ETN	11	Tumefactive demyelinating lesions	Brain gd +	No	Yes	PLEX, craniectomy	No	No	6
87	M/34	NR	AS HLAB27+	NSAIDs	ETN	30	MDE	Brain, CSC gd-	NR	Yes	None	No	New gd + lesion in 10 m, MS diagnosis	10
88	M/32	NR	CD, HLAb27- bilateral sacroilitis	AZA, MES, oral steroids	IFX, oral steroids	1,5	MS	Brain, SC gd-		Yes	INFb, AZA, PREDN	Complete	No	6
89	M/55	No	CD	AZA, MES	IFX	10	ON	NR	NR	Yes	Pulse steroids	Partial	No	2
90	F/39	NR	RA	NR	IFX, MTX	0,1	ON	Brain gd+	NR	Yes	Pulse steroids	Partial	No	5
91	F/51	NR	UC	MES, PREDN, AZA	IFX	17	ON	Brain gd+	NR	Yes	Pulse steroids	Complete	No	1
92	M/68	NR	CD	NR	IFX	1,5	ON bilateral	Brain nm	No	Yes	Pulse steroids	No	No	5
93	F/44	NR	PsA	NR	Golimumab + LEFL		Tumefactive lesions	Brain gd +	Yes	Yes	Pulse steroids	Partial	Later MS diagnosis and therapy with dimethyl fumarate	16
94	M/35	NR	PsA	NR	ETN, IFX	IFX 8	MDE, peroneal palsy	Brain, CSC gd-	NR	Yes	Pulse steroids	Complete (2 m)	NR	2
94	F/45	NR	RA	NR	ADM	6	ON	Brain nm	NR	Yes	None	Complete (2 m)	Flare of RA – restarted ADM – second ON	4
Index case 1	M/17	No	PsA	NR	ETN	8	MS	Brain, SC gd+	Yes	Yes	Pulse steroids	Complete	MRI progression, MS relapses, PsA progression (CYC)	48
Index case 2	M/27	Yes	AS	NSAIDs	ADM	36	MS	Brain, CSC gd +	Yes	Yes	Pulse steroids	Complete	MRI progression, AS progression (no treatment)	48
Index case 3	F/46	No	PsA	NR	ETN	48	MDE	Brain, CSC gd -	Yes	Yes	Pulse steroids	Complete	No PsA progresson -HCQ	48
Index case 4	F/57	No	AS	NR	ETN	72	MS	Brain, CSC gd+	Yes	Yes	Pulse steroids	Complete	No	36
REVIEWS														
citation		N	Sex (n)	Mean age in years (SD)	Diagnosis (n)	antiTNFa agent (n)	Mean exposure time (months)		Type of demyelination (n)		Therapy		Recovery	Mean follow-up (months)
30		20	F12/M 8	43,5 (9,27)	RA (11), PsA (4), JRA (2), other (3)	IFX (2), ETN (18)	5		MS (4), ON (7), MDE (10), other (4)		Pulse streroids (4), oral steroids (4), IVIG (2), PLEX (1), lfuoxetine (1), INFb-glatiramer acetate (1), NR (8)		Complete (4), partial (6), no (4), NR (5)	4
95	BIOBADASER	14	F 9/ M 5	51 (11)	RA (9), AS (2), PsA (3)	IFX (8), ETN (5), ADM (1)	17		MS (1), ON (4), MDE (9)		Steroids (3), none (3), INFb (1)		Complete (5), partial (1), no (7), NR (1)	NR
95	FEDRA	19	F 10/ M 9	NR	RA (10), AS (3), PsA (4), other (2)	IFX (11), ETN (6), ADM (2)	12		MS (9), ON (8), MDE (2)		NR		Complete (7), no (8), NR (4)	NR

*F* female, *M* male, *mo* months, *y* years, *wk* weeks, *d* days, *CD* Crohn’s disease, *RA* rheumatoid arthritis, *Ps* psoriasis, *PsA* psoriatic arthritis, *UC* ulcerative colitis, *CIAU* chronic idiopathic non-granulomatous anterior uveitis, *NLD* necrobiosis lipoidica diabeticorum, *IFX* infliximab, *ETN* etanercept, *ADM* adalimumab, *ON* optic neuritis, *MS* multiple sclerosis, *PML* progressive multifocal leukoencephalopathy, *MDE* monophasic demyelinating event, *SUL* sulfasalazine, *LEFL* leflunomide, *PREDN* prednisolone, *HCQ* hydroxychloroquine, *NSAIDs* non-steroidal anti-inflammatory drugs, *AZA* azathioprine, *MES* mesalazine, *CYC* cyclophosphamide, *6-MP* 6-mercaptopurine: MTX: methotrexate, *MITO* mitoxandrone, *GST* gold sodium thiomalate, *D-P* d-penicillamine, *Buc* bucillamine, *Sal* salazosulfapyridine, *NR* not rated, *gd* gadolinium enhancement ±, *SC* spinal cord, *SCC* cervical spinal cord, *nm* normal, *TM* transverse myelitis, *PN* peripheral neuropathy, *NR* not reported, *TNF* tumor necrosis factor, *UA* unilateral, *IVIG* intravenous immunoglobulin, *OCB* oligoclonal bands, *PLEX* plasma exchange.

## Our Experience

**Fig. 1:**
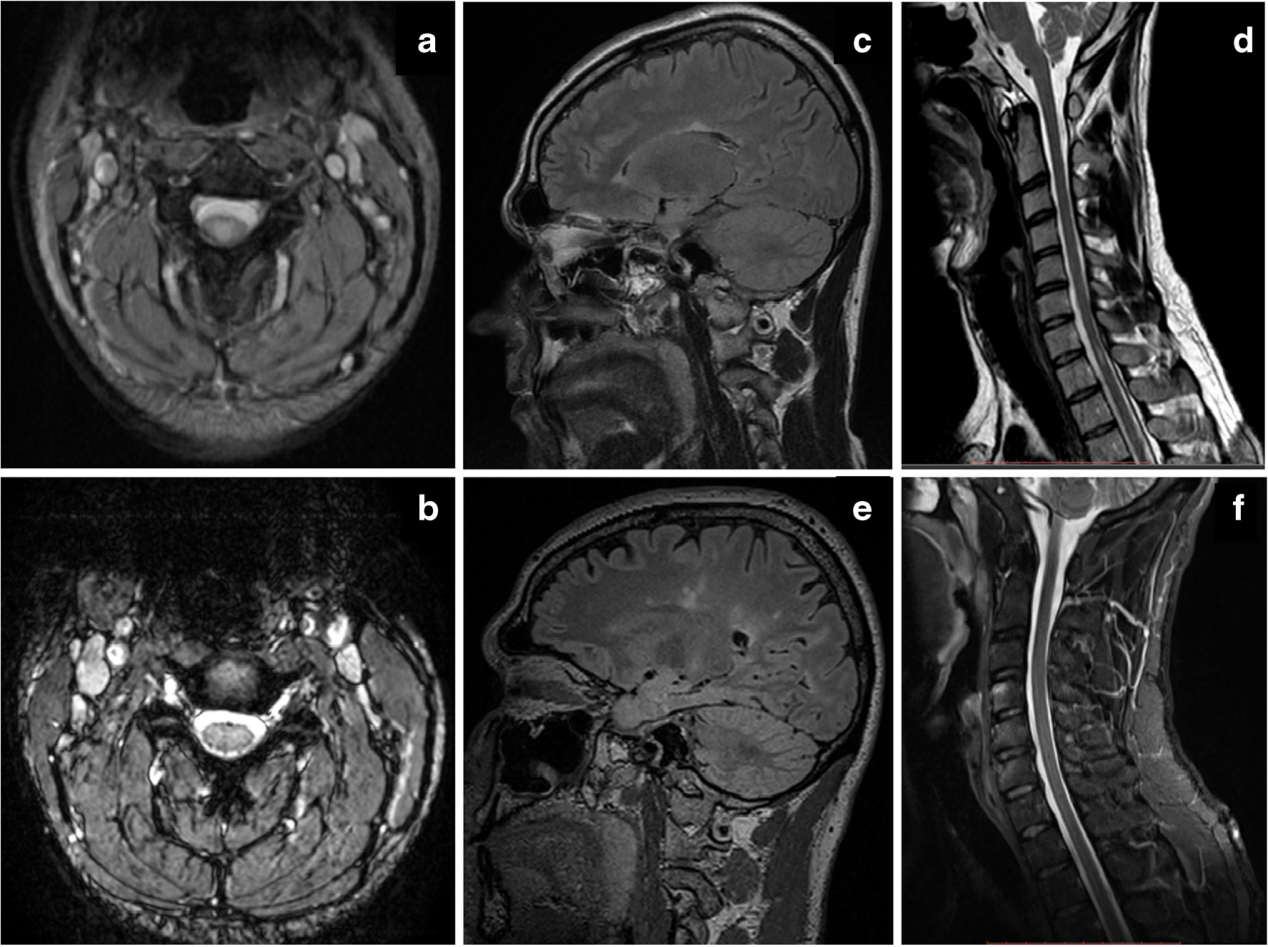
**(a)** and **(b)**: axial T2-weighted cervical spine MRI of the index case 1, treated with ETN for 8 months for PsA, who was diagnosed with MS on follow-up. **(a)** At initial presentation, a central posterior demyelinating lesion on C3 level that showed gadolinium enhancement on T1-sequences (not shown). **(b)** Four years after etanecept discontinuation, disappearance of the lesion. **(c)**–**(f)** Brain and cervical spine MRI of the index case 2, with AS and a family history of MS, treated with adalimumab for 36 months, who was also diagnosed with MS. **(c)** Brain flair and **(d)** cervical spine T2-weighted sagittal MRI at symptom onset, and **(e)**–**(f)** 4 years after anti-TNF-a cessation. Note the increase of cerebral demyelinating lesions, with marked atrophy, and the disappearance of the lesion on C7 level

Our patients had been treated with TNF-α blockers previously or were taking them at the onset of symptoms; thus, a correlation between the treatment and demyelination was speculated. Anti-TNF-α treatment was discontinued as soon as demyelination was suspected and steroids were administered intravenously with consequent clinical improvement. During the 5-year follow-up, 2 patients remained stable without relapses or new MRI lesions, whereas the other 2, both male, had clinical relapses and radiological deterioration with new brain and spinal cord (SC) demyelinating lesions. One of them required pulsed steroid treatment, whereas the other had a minor relapse that subsided without therapy (Fig. 1). It is remarkable that both these patients had a family history of autoimmune diseases, a fact that could indicate an increased susceptibility of CNS demyelination, irrespectively of anti-TNF-α treatment [35] and should be taken into consideration during the clinical evaluation of the patients. On the other hand, anti-TNF-α agents could potentially aggravate demyelination in genetically predisposed patients (such as first degree relatives of MS patients as in our second case). Therefore, the assumption that in our cases treatment might have unmasked pre-existing latent MS seems plausible. Nevertheless, such an association could not be established, since neurologic examination or brain MRI had not been performed prior to anti-TNF-α treatment initiation.

None of our patients received specific MS treatment, although all of them were treated with immunesuppressants for their initial autoimmune disease (AS or PsA), after the presenting demyelinating event.

## Outcome of Rheumatic Disease After TNF-α Blocker Discontinuation

TNF-α blockers are currently recommended for patients not responding to at least 2 different DMDs and seem to have an excellent and sustained therapeutic outcome: 80% of patients improve rapidly and 50% have complete remission [94, 95]. Patients who receive TNF-α blockers, therefore, are refractory to standard medical therapy. Unfortunately, most reports of demyelinating events after administration of TNF-α blockers focus on the clinical evolution of demyelination and do not provide information on the course of the underlying rheumatic or inflammatory disease. Based on the data collected, it appears that the rheumatic/inflammatory disease follows an independent course and most patients continue treatment with DMDs, often with poor results [71].

## Outcome of CNS Demyelination Associated with TNF-α Blockers

Although CNS demyelination after treatment with TNF-α blockers is not necessarily associated with the duration of the therapy and drug discontinuation does not always lead to improvement [93], treatment should be discontinued at the appearance of unexplained neurologic symptoms. On the other hand, there are no clear recommendations for the management of the CNS demyelinating process. In most cases, the therapeutic regimen is based on the standard guidelines for CNS demyelination. Steroids are usually administered, either pulses of methylprednisolone or oral glucocorticosteroids, with good results in the short term, although the course of the demyelinating disease appears unpredictable [1, 30].

## Arguments for a Possible Relationship Between Anti-TNF-α Therapy and Demyelination

### Several Factors Suggest an Etiological Role of TNF-α Blockers in CNS Demyelination

1. Reported cases and pathogenic theories

The large number of CNS and peripheral demyelinating disorders after TNF-α blocker administration published in the literature [2, 30, 49–54, 55•, 56, 57••, 58–90, 91•, 92••, 93] and the 2 clinical trials of TNF-α blockers in MS patients showing an increase of demyelinating events [22, 23] raise the question of a possible association. Moreover, many theories support a possible correlation of non-selective TNF-α blockade with demyelination.

2. Miller criteria

Some of the published cases meet the Miller criteria [96], attributing the CNS demyelinating events to the TNF-a blockade. According to Miller criteria, the definition of drug-induced illness comprises 8 elements and requires the presence of at least 4: temporal association, improvement of symptoms after treatment discontinuation, positive rechallenge phenomenon, and other CNS demyelinating events reported in the literature [96]. In most cases, a temporal relation to anti-TNF-α treatment is suggested [72], with resolution of symptoms after treatment discontinuation [27, 97]. Few cases show a rechallenge phenomenon and even fewer argue in favor of the onset of a demyelinating process directly after anti-TNF-α exposure [67].

3. Age of onset

The peak age of MS onset is 20–40 years old, whereas the mean age of RA onset is 40–60 years old. In our review, the mean age of the patients who developed CNS demyelination was 45.47 years. This delayed onset of demyelination could suggest a possible association with anti-TNF-α use.

## Arguments Against a Possible Relationship Between TNF-α Blockers and Demyelination

Several factors, on the other hand, challenge a causal relationship of anti-TNF-α treatment with the appearance of CNS demyelination

1. Incidence

Although over 2 million patients with rheumatic and other chronic inflammatory diseases have been treated with anti-TNF-α therapies [98••], the overall number of the published demyelinating events is relatively small. In initial reports, the estimated risk of CNS demyelination after anti-TNF-α therapy ranged widely between 30% and that of the general population [30, 58, 69]. Interestingly, randomized controlled trials, developmental and post-marketing programs, and retrospective long-term safety studies revealed no increase of demyelinating events compared with the actual incidence of MS [27, 99, 100]. A Danish population-based cohort study of IBD patients exposed to TNF-α blockers showed a 2-fold increase in relative risk, but a low absolute risk of CNS demyelination.

2. However, it should be emphasized that demyelinating events could be silent and therefore under-reported, making it difficult to estimate the actual incidence of CNS demyelination after anti-TNF-α therapy [75]. This issue was addressed in a prospective study of patients with rheumatic diseases before receiving TNF-α blockers. Two out of 77 patients had MRI lesions and 4 developed demyelination, but the overall rate of neurologic adverse events was comparable with the group of patients who had not received anti TNF -α blockers [92••]. Autoimmunity

Patients with autoimmune diseases could be genetically prone to develop another autoimmune disease [101], a fact that might predispose them to CNS demyelination regardless of anti-TNF-α use. On the other hand, the use of such therapies could merely unmask latent MS [53]. Moreover, demyelination could be part of an overlapping syndrome with other rheumatologic diseases. Although RA patients showed no increase in latent demyelination [97], IBD patients appeared to have a high occurrence of CNS T2 white matter lesions and a higher risk of developing CNS demyelination [77, 102–105]. Despite the fact that several case reports revealed a higher prevalence of RA, psoriasis, and goiter in MS patients [52–54, 55•, 56, 57••, 58], no further accumulation of autoimmune diseases was found in a multi-center population-based study [106].

3. Previous treatments

TNF-α blockers are therapeutic options for patients who have already received treatment with a number of other immunosuppressive agents, most commonly methotrexate. Although methotrexate is an immunosuppressant extensively used in MS, it has been associated with demyelinating events at high doses and in combination with cranial radiotherapy [107].

4. Rechallenge phenomenon

The rechallenge phenomenon could be simply explained by the relapsing-remitting nature of MS [108]. On the other hand, most of the patients who were retreated with TNF-α blockers after a demyelinating event did not experience new neurologic symptoms.

5. Time interval between therapy and symptom onset

The interval between treatment administration and symptom initiation varies greatly, from 5 months up to 4 years [30]. In the presently reviewed cases, symptoms appeared after a mean exposure time of 17.61 months. However, given that the anti-TNF-α effect could last longer than its half-life, resulting in delayed side effects [1], symptoms may even emerge after treatment cessation [30].

6. Outcome

In most published cases, demyelination was either slowly progressing or stopped after anti-TNF-α discontinuation. This could suggest that anti-TNF-α therapy exerts a protective effect in patients already suffering from latent MS, or has a short lasting harmful impact.

## Conclusions

Anti-TNF-α blockers have reshaped the treatment of rheumatoid and autoimmune diseases, being the most effective drugs in our therapeutic armentarium. However, TNF-α functioning, especially in the CNS, is still incompletely understood, setting restrictions on their unlimited use. To date, the reported cases of demyelination address the possibility of a causal association. Our previous study raised the question whether the demyelinating events in patients who had previously received TNF-α blockers were the result of uncovering latent MS, onset of a novel demyelinating event (MS or MS-like syndrome), or were merely an accidental coexistence of the 2 disorders. Long-term follow-up of these patients is required to properly diagnose, determine the clinical course, and point out the potential differences from typical MS. Complete symptom remission after therapy withdrawal or a positive rechallenge phenomenon could prove causality, although the nature of MS, with relapses and remissions of symptoms, argues against such a relationship. The complexity of the biological functions of TNF-α further complicates the issue. Although several theoretical explanations of demyelination after TNF-α blockade have been proposed, the relation of these events with the use of TNF-α blockers remains to be elucidated.

Nevertheless, according to the current guidelines, avoiding the use of anti-TNF-α therapy is recommended in patients with a history or familial occurrence of MS or other demyelinating diseases. Furthermore, in case of appearance of neurologic symptoms, the patient should undergo a thorough neurologic assessment (level of evidence III and a grade of recommendation B) [108]. Besides those with a family history of CNS demyelination, patients with familial occurrence of systemic autoimmune diseases (as our first case) might also be at increased risk of CNS demyelination with anti-TNF-α treatment. Although MRI imaging of the brain before starting anti-TNF-α treatment is not recommended [108], it could be useful in revealing possible silent demyelinating lesions [56], especially in patients with a family history of MS or systemic autoimmune diseases.

We propose careful clinical evaluation of candidates for TNF-α blockers, including neurologic assessment before drug initiation and close neurologic monitoring, in order to diagnose a possible demyelinating condition in as timely a manner as possible, especially in high risk patients.

## Future Prospects

Anti-TNF-α therapies undoubtedly are an excellent therapeutic choice in refractory inflammatory diseases. On the other hand, considering the possibility of demyelination, more specific TNF-α-targeting drugs might be safer. Anti-TNF-α therapies that either target TNFR1 while sparing TNFR2 signaling selectively, or inhibit sTNF (currently under investigation) could potentially minimize the adverse events in the CNS. Furthermore, based on the observation that TNFR2 antagonizes TNFR1 signaling, either TNFR2 agonists or increased expression of TNFR2 by gene therapy might be beneficial [108]. The possibility of a more susceptible patient population that should be cautiously treated with TNF-α blockers must be taken into account, and in these cases the detection of a potential predictive marker could be helpful.

Whether TNF-α blockers directly cause demyelination (either with a progressive or a monophasic course) or they trigger pre-existing demyelinating predisposition, still remains controversial. Unfortunately, only few patients with a satisfactory follow-up are reported in the literature. Systematic and long-term follow-up of affected patients might shed light to these still unanswered questions and help distinguish which patients will safely benefit from anti-TNF-α treatment for their inflammatory disease. Furthermore, elucidation of the exact mechanism of the apparently paradoxical response to TNF-α blockers in MS might improve our understanding of the pathophysiology of MS.

## References

[1] Bosch X, Ramos-Casals M (2011). Monoclonal antibody therapy-associated neurologic disorders. Nat Rev Neurol.

[2] Andreadou E, Kemanetzoglou E, Brokalaki C, Evangelopoulos M, Kilidireas C, Rombos A, et al. Demyelinating disease following anti-TNF-α treatment: a causal or coincidental association? Report of Four cases and review of the literature. Case Rep Neurol Med. 2013;671935. 10.1155/2013/671935 PMC3670521 23762678

[3] Robinson W, Genovese M, Moreland L (2001). Demyelinating and neurologic events reported in association with tumor necrosis factor α antagonism. Arthritis Rheum.

[4] Kollias G, Douni E, Kassiotis G, Kontoyiannis D (1999). The role of tumor necrosis factor and receptors in models of multi organ inflammation, rheumatoid arthritis, multiple sclerosis, and inflammatory bowel disease. Immunol Rev.

[5] Caminero A, Comabella M, Montalban X (2011). Tumor necrosis factor α (TNF-α), anti-TNF-α and demyelination revisited: an ongoing story. J Neuroimmunol.

[6] Kassiotis G, Kollias G (2001). Uncoupling the proinflammatory from the immunosuppressive properties of tumor necrosis factor (TNF) at the p55 TNF receptor level: implications for pathogenesis and therapy of autoimmune demyelination. J Exp Med.

[7] Lim S, Constantinescu C, Lim SY, Constantinescu CS (2010). TNF-α: a paradigm of paradox and complexity in multiple sclerosis and its animal models. Open Autoimmun J.

[8] Brambilla R, Ashbaugh J, Magliozzi R, Dellarole A, Karmally S, Szymkowski D (2011). Inhibition of soluble tumour necrosis factor is therapeutic in experimental autoimmune encephalomyelitis and promotes axon preservation and remyelination. Brain.

[9] Probert L (2015). TNF and its receptors in the CNS: the essential, the desirable and the deleterious effects. Neuroscience.

[10] Kalliolias G, Ivashkiv L (2016). TNF biology, pathogenic mechanisms, and emerging therapeutic strategies. Nat Rev Rheumatol.

[11] Thalayasingam N, Isaacs J (2011). Anti-TNF therapy. Best Pract Res Clin Rheumatol.

[12] Weiss G, Schaible U (2015). Macrophage defense mechanisms against intracellular bacteria. Immunol Rev.

[13] Linkermann A, Stockwell B, Krautwald S, Anders H (2014). Regulated cell death and inflammation: an auto-amplification loop causes organ failure. Nat Rev Immunol.

[14] Nishioku T, Matsumoto J, Dohgu S, Sumi N, Miyao K, Takata F (2010). Tumor necrosis factor-α mediates the blood-brain barrier dysfunction induced by activated microglia in mouse brain microvascular endothelial cells. J Pharmacol Sci.

[15] Horiuchi T, Mitoma H, Harashima S, Tsukamoto H, Shimoda T (2010). Transmembrane TNF-αlpha: structure, function, and interaction with anti-TNF agents. Rheumatology (Oxford).

[16] Silva L, Ortigosa L, Benard G (2010). Anti-TNF-α agents in the treatment of immune-mediated inflammatory diseases: mechanisms of action and pitfalls. Immunotherapy.

[17] Scallon B, Cai A, Solowski N, Song X, Song X, Shealy D (2002). Binding and functional comparisons of two types of tumor necrosis factor antagonists. J Pharmacol Exp Ther.

[18] Tristano A (2010). Neurologic adverse events associated with anti-tumor necrosis factor α treatment. J Neurol.

[19] Machado M, Barbosa M, Almeida A, de Araújo V, Kakehasi A, Andrade E (2013). Treatment of ankylosing spondylitis with TNF blockers: a meta-analysis. Rheumatol Int.

[20] Alexis A, Strober B (2005). Off-label dermatologic uses of anti-TNF-α therapies. J Cutan Med Surg.

[21] Sánchez-Cano D, Callejas-Rubio J, Ruiz-Villaverde R, Ríos-Fernández R, Ortego-Centeno, Ortego-Centeno N. Off-label uses of anti-TNF therapy in three frequent disorders: Behçet's disease, sarcoidosis, and noninfectious uveitis. Mediators Inflamm. 2013;286857. 10.1155/2013/286857 PMC3747407 23983404

[22] van Oosten B, Barkhof F, Truyen L, Boringa J, Bertelsmann F, von Blomberg B (1996). Increased MRI activity and immune activation in two multiple sclerosis patients treated with the monoclonal anti-tumor necrosis factor antibody cA2. Neurology.

[23] The Lenercept Multiple Sclerosis Study Group and The University of British Columbia MS/MRI Analysis Group TNF neutralization in MS: results of a randomized, placebo-controlled multicenter study. Neurology. 1999;53(3):457–65. 10449104

[24] Committee, British Thoracic Society Standards of Care (2005). BTS recommendations for assessing risk and for managing *Mycobacterium tuberculosis* infection and disease in patients due to start anti-TNF-αlpha treatment. Thorax.

[25] Strangfeld A, Listing J (2006). Infection and musculoskeletal conditions: bacterial and opportunistic infections during anti-TNF therapy. Best Pract Res Clin Rheumatol.

[26] Brown A, Staugaitis S, Wu A, Calabrese L. Central nervous system vasculitis complicating rheumatoid arthritis in a patient on a TNF inhibitor: a causal association? Case report and systematic review. J Vasc. 2:104.

[27] Ramos-Casals M, Brito-Zerón P, Muñoz S, Soria N, Galiana D, Bertolaccini L (2007). Autoimmune diseases induced by TNF-targeted therapies: analysis of 233 cases. Medicine (Baltimore).

[28] Zengin O, Onder M, Alkan S, Kimyon G, Hüseynova N, Demir Z, et al. Three cases of anti-TNF induced myositis and literature review. Rev Bras Reumatol. 2016. (English edition) Mar 6. 10.1016/j.rbre.2016.05.003 29173693

[29] Bruzzese V, Marrese C, Scolieri P, Hassan C, Lorenzetti R, Zullo A (2015). Myasthenia gravis onset during rheumatic disease: a new paradoxical effect of anti-TNF-α therapy?. Int J Rheum Dis.

[30] Mohan N, Edwards E, Cupps T, Oliverio P, Sandberg G, Crayton H (2001). Demyelination occurring during anti-tumor necrosis factor α therapy for inflammatory arthritides. Arthritis Rheum.

[31] Körner H, Riminton DS, Strickland DH, Lemckert FA, Pollard JD, Sedgwick JD (1997). Critical points of tumor necrosis factor action in central nervous system autoimmune inflammation defined by gene targeting. J Exp Med.

[32] Hofman FM, Hinton DR, Johnson K, Merrill JE (1989). Tumor necrosis factor identified in multiple sclerosis brain. J Exp Med.

[33] Sharief MK, Hentges R (1991). Association between tumor necrosis factor-α and disease progression in patients with multiple sclerosis. N Engl J Med.

[34] Maimone D, Gregory S, Arnason BG, Reder AT (1991). Cytokine levels in the cerebrospinal fluid and serum of patients with multiple sclerosis. J Neuroimmunol.

[35] Rieckmann P, Albrecht M, Kitze B, Weber T, Tumani H, Broocks A (1995). Tumor necrosis factor-α messenger RNA expression in patients with relapsing-remitting multiple sclerosis is associated with disease activity. Ann Neurol.

[36] van Oosten BW, Barkhof F, Scholten PE, von Blomberg BM, Adèr HJ, Polman CH (1998). Increased production of tumor necrosis factor-α, and not of interferon-γ, preceding disease activity in patients with multiple sclerosis. Arch Neurol.

[37] Valentin-Torres A, Savarin C, Hinton DR, Phares TW, Bergmann CC, Stohlman SA (2016). Sustained TNF production by central nervous system infiltrating macrophages promotes progressive autoimmune encephalomyelitis. J Neuroinflamm.

[38] Kroner A, Greenhalgh AD, Zarruk JG, Passos Dos Santos R, Gaestel M, David S (2014). TNF and increased intracellular iron alter macrophage polarization to a detrimental M1 phenotype in the injured spinal cord. Neuron.

[39] Kruglov AA, Lampropoulou V, Fillatreau S, Nedospasov SA (2011). Pathogenic and protective functions of TNF in neuroinflammation are defined by its expression in T lymphocytes and myeloid cells. J Immunol.

[40] Arnett HA, Mason J, Marino M, Suzuki K, Matsushima GK, Ting J (2001). TNF-α promotes proliferation of oligodendrocyte progenitors and remyelination. Nat Neurosci.

[41] Ruddle NH, Bergman CM, McGrath KM, Lingenheld EG, Grunnet ML, Padula SJ (1990). An antibody to lymphotoxin and tumor necrosis factor prevents transfer of experimental allergic encephalomyelitis. J Exp Med.

[42] Murphy CA, Hoek RM, Wiekowski MT, Lira SA, Sedgwick JD (2002). Interactions between hemopoietically derived TNF and central nervous system-resident glial chemokines underlie initiation of autoimmune inflammation in the brain. J Immunol.

[43] Baker D, Butler D, Scallon BJ, O'Neill JK, Turk JL, Feldmann M (1994). Control of established experimental allergic encephalomyelitis by inhibition of tumor necrosis factor (TNF) activity within the central nervous system using monoclonal antibodies and TNF receptor-immunoglobulin fusion proteins. Eur J Immunol.

[44] Alexopoulou L, Kranidioti K, Xanthoulea S, Denis M, Kotanidou A, Douni E (2006). Transmembrane TNF protects mutant mice against intracellular bacterial infections, chronic inflammation, and autoimmunity. Eur J Immunol.

[45] Gregory AP, Dendrou CA, Attfield KE, Haghikia A, Xifara DK, Butter F (2012). TNF receptor 1 genetic risk mirrors outcome of anti-TNF therapy in multiple sclerosis. Nature.

[46] Sedger LM, McDermott MF (2014). TNF, and TNF-receptors: from mediators of cell death and inflammation to therapeutic giants—past, present, and future. Cytokine Growth Factor Rev.

[47] Kaltsonoudis E, Voulgari PV, Konitsiotis S, Drosos AA (2014). Demyelination and other neurologic adverse events after anti-TNF therapy. Autoimmun Rev.

[48] Cisternas M, Gutiérrez M, Jacobelli S (2002). Successful rechallenge with anti-tumor necrosis factor-α for psoriatic arthritis after development of demyelinating nervous system disease during initial treatment: comment on the article by Mohan et al. Arthritis Rheum.

[49] Titelbaum DS, Degenhardt A, Kinkel R (2005). Anti-tumor necrosis factor-α-associated multiple sclerosis. AJNR Am J Neuroradiol.

[50] Ruiz-Jimeno T, Carvajal A, Mata C, Aurrecoechea E (2006). Demyelinating disease in a patient with psoriatic arthritis and family history of multiple sclerosis treated with infliximab. J Rheumatol.

[51] Dubcenco E, Ottaway CA, Chen DL, Baker J (2006). Neurologic symptoms suggestive of demyelination in Crohn's disease after infliximab therapy. Eur J Gastroenterol Hepatol.

[52] Toussirot E, Pertuiset E, Martin A, Melac-Ducamp S, Alcalay M, Grardel B (2006). Association of rheumatoid arthritis with multiple sclerosis: report of 14 cases and discussion of its significance. J Rheumatol.

[53] Noseworthy JH, Lucchinetti C, Rodriguez M, Weinshenker BG (2000). Multiple sclerosis. N Engl J Med.

[54] van Boxel-Dezaire AH, Hoff SC, van Oosten BW, Verweij CL, Dräger AM, Adèr HJ (1999). Decreased interleukin-10 and increased interleukin-12p40 mRNA are associated with disease activity and characterize different disease stages in multiple sclerosis. Ann Neurol.

[55] Miller PG, Bonn MB, Franklin CL, Ericsson AC, McKarns SC (2015). TNFR2 deficiency acts in concert with gut microbiota to precipitate spontaneous sex-biased central nervous system demyelinating autoimmune disease. J Immunol.

[56] Bellesi M, Logullo F, Di Bella P, Provinciali L (2006). CNS demyelination during anti-tumor necrosis factor α therapy. J Neurol.

[57] Mausner-Fainberg K, Regev K, Kolb H, Vaknin-Dembinsky A, Karni A (2015). Increased neutralization capacity of TNF-α in sera of relapsing remitting multiple sclerosis patients is not related to soluble TNF-α receptors or anti-TNF-α autoantibody levels. J Neuroimmunol.

[58] Bernatsky S, Renoux C, Suissa S (2010). Demyelinating events in rheumatoid arthritis after drug exposures. Ann Rheum Dis.

[59] Voulgari PV, Alamanos Y, Nikas SN, Bougias DV, Temekonidis TI, Drosos AA (2005). Infliximab therapy in established rheumatoid arthritis: an observational study. Am J Med.

[60] Simsek I, Erdem H, Pay S, Sobaci G, Dinc A (2007). Optic neuritis occurring with anti-Tumor Necrosis Factor-α therapy. Ann Rheum Dis.

[61] Bruè C, Mariotti C, Rossiello I, Saitta A, Giovannini A (2016). Demyelinizing neurologic disease after treatment with tumor necrosis factor-α antagonists. Case Rep Ophthalmol.

[62] Defty H, Sames E, Doherty T, Hughes R (2013). Case report of transverse myelitis in a patient receiving etanercept for rheumatoid arthritis. Case Rep Rheumatol.

[63] Ryu YS, Park SH, Kim JM, Kim EJ, Lee J, Kwok SK (2012). A case of leukoencephalopathy associated with adalimumab-treated rheumatoid arthritis and a review of literature. Rheumatol Int.

[64] Kameda T, Dobashi H, Kittaka K, Susaki K, Hosomi N, Deguchi K (2008). A case of rheumatoid arthritis complicated by demyelination in both cerebral cortex and spinal cord during etanercept therapy. Mod Rheumatol.

[65] Bensouda-Grimaldi L, Mulleman D, Valat JP, Autret-Leca E (2007). Adalimumab-associated multiple sclerosis. J Rheumatol.

[66] Matsumoto T, Nakamura I, Miura A, Momoyama G, Ito K (2013). New-onset multiple sclerosis associated with adalimumab treatment in rheumatoid arthritis: a case report and literature review. Clin Rheumatol.

[67] Tanno M, Nakamura I, Kobayashi S, Kurihara K, Ito K (2006). New-onset demyelination induced by infliximab therapy in two rheumatoid arthritis patients. Clin Rheumatol.

[68] Al Saieg N, Luzar MJ (2006). Etanercept induced multiple sclerosis and transverse myelitis. J Rheumatol.

[69] Pfueller CF, Seipelt E, Zipp F, Paul F (2008). Multiple sclerosis following etanercept treatment for ankylosing spondylitis. Scand J Rheumatol.

[70] Kunzmann S, Warmuth-Metz M, Girschick HJ (2005). Cerebral demyelination in association with TNF-inhibition therapy in a 5-year-old girl with aseptic meningitis as the first symptom of Still's disease. Scand J Rheumatol.

[71] Fromont A, De Seze J, Fleury MC, Maillefert JF, Moreau T (2009). Inflammatory demyelinating events following treatment with anti-tumor necrosis factor. Cytokine.

[72] Kim A, Saffra N (2012). A case report of adalimumab-associated optic neuritis. J Ophthalmic Inflamm Infect.

[73] Faillace C, de Almeida JR, de Carvalho JF (2013). Optic neuritis after infliximab therapy. Rheumatol Int.

[74] Davis SA, Johnson RR, Pendleton JW (2008). Demyelinating disease associated with use of etanercept in patients with seronegative spondyloarthropathies. J Rheumatol.

[75] Gomez-Gallego M, Meca-Lallana J, Fernandez-Barreiro A (2008). Multiple sclerosis onset during etanercept treatment. Eur Neurol.

[76] Enayati PJ, Papadakis KA (2005). Association of anti-tumor necrosis factor therapy with the development of multiple sclerosis. J Clin Gastroenterol.

[77] Solomon AJ (2011). SRKMBD. Inflammatory neurologic disease in patients treated with tumor necrosis factor-α blockers. Mult Scler.

[78] Winkelmann A, Patejdl R, Wagner S, Benecke R, Zettl UK (2008). Cerebral MRI lesions and anti-tumor necrosis factor-α therapy. J Neurol.

[79] Hare NC, Hunt DP, Venugopal K, Ho GT, Beez T, Lees CW (2014). Multiple sclerosis in the context of TNF blockade and inflammatory bowel disease. QJM.

[80] Bradshaw MJ, Mobley BC, Zwerner JP, Sriram S (2016). Autopsy-proven demyelination associated with infliximab treatment. Neurol Neuroimmunol Neuroinflamm.

[81] Molloy ES, Calabrese LH (2012). Progressive multifocal leukoencephalopathy associated with immunosuppressive therapy in rheumatic diseases: evolving role of biologic therapies. Arthritis Rheum.

[82] Kumar D, Bouldin TW, Berger RG (2010). A case of progressive multifocal leukoencephalopathy in a patient treated with infliximab. Arthritis Rheum.

[83] Graff-Radford J, Robinson MT, Warsame RM, Matteson EL, Eggers SD, Keegan BM (2012). Progressive multifocal leukoencephalopathy in a patient treated with etanercept. Neurologist.

[84] Cereda CW, Zecca C, Mazzucchelli L, Valci L, Staedler C, Bassetti CL (2013). Tumefactive demyelinating lesions during etanercept treatment requiring decompressive hemicraniectomy. Mult Scler.

[85] Mercieca C, Vella N, Borg AA (2012). Demyelination during anti-TNF-α therapy for ankylosing spondylitis. Mod Rheumatol.

[86] Felekis T, Katsanos K, Christodoulou D, Asproudis I, Tsianos EV (2009). Reversible bilateral optic neuritis after infliximab discontinuation in a patient with Crohn's disease. J Crohns Colitis.

[87] Ouakaa-Kchaou A, Gargouri D, Trojet S, Hefaiedh R, Elloumi H, Kochlef A (2009). Retrobulbar optic neuritis associated with infliximab in a patient with Crohn's disease. J Crohns Colitis.

[88] Pikkel J (2008). Possible link between infliximab and optic neuritis. Isr Med Assoc J.

[89] Mumoli N, Niccoli G, Scazzeri F, Picchietti S, Greco A, Cei M (2007). Infliximab-induced retrobulbar optic neuritis. QJM.

[90] Chan JW, Castellanos A (2010). Infliximab and anterior optic neuropathy: case report and review of the literature. Graefes Arch Clin Exp Ophthalmol.

[91] Maillart E, Papeix C, Mellerio C, Bertrand A, Lubetzki C, Louapre C (2016). Extensive and severe CNS demyelination associated with golimumab therapy. J Neurol.

[92] Kaltsonoudis E, Zikou AK, Voulgari PV, Konitsiotis S, Argyropoulou MI, Drosos AA (2014). Neurologic adverse events in patients receiving anti-TNF therapy: a prospective imaging and electrophysiological study. Arthritis Res Ther.

[93] Cruz Fernández-Espartero M, Pérez-Zafrilla B, Naranjo A, Esteban C, Ortiz AM, Gómez-Reino JJ (2011). Demyelinating disease in patients treated with TNF antagonists in rheumatology: data from BIOBADASER, a pharmacovigilance database, and a systematic review. Semin Arthritis Rheum.

[94] Hanauer SB, Feagan BG, Lichtenstein GR, Mayer LF, Schreiber S, Colombel JF (2002). Maintenance infliximab for Crohn’s disease: the ACCENT I randomised trial. Lancet.

[95] Schnitzler F, Fidder H, Ferrante M, Noman M, Arijs I, Van Assche G (2009). Long-term outcome of treatment with infliximab in 614 patients with Crohn’s disease: results from a single-centre cohort. Gut.

[96] Miller FW, Hess EV, Clauw DJ, Hertzman PA, Pincus T, Silver RM (2000). Approaches for identifying and defining environmentally associated rheumatic disorders. Arthritis Rheum.

[97] Ramos-Casals M, Roberto-Perez-Alvarez, Diaz-Lagares C, Cuadrado MJ, Khamashta MA, BIOGEAS Study Group (2010). Autoimmune diseases induced by biological agents: a double-edged sword?. Autoimmun Rev.

[98] •• Nyboe Andersen N, Pasternak B, Andersson M, Nielsen NM, Jess T. Risk of demyelinating diseases in the central nervous system in patients with inflammatory bowel disease treated with tumor necrosis factor blockers. JAMA Intern Med. 2015;175(12):1990–2. **This population based cohort study by Andersen et al, used the Danish Civil Registration System in order to identify IBD patients exposed to anti-TNF-α patients (4504) and matched unexposed patients (16.429). A possible 2-fold relative risk but a low absolute risk of CNS demyelination was suggested**. 10.1001/jamainternmed.2015.5396 26437461

[99] Schiff MH, Burmester GR, Kent JD, Pangan AL, Kupper H, Fitzpatrick SB (2006). Safety analyses of adalimumab (HUMIRA) in global clinical trials and US post-marketing surveillance of patients with rheumatoid arthritis. Ann Rheum Dis.

[100] Lees CW, Ali AI, Thompson AI, Ho GT, Forsythe RO, Marquez L (2009). The safety profile of anti-tumor necrosis factor therapy in inflammatory bowel disease in clinical practice: analysis of 620 patient-years follow-up. Aliment Pharmacol Ther.

[101] Nielsen NM, Frisch M, Rostgaard K, Wohlfahrt J, Hjalgrim H, Koch-Henriksen N (2008). Autoimmune diseases in patients with multiple sclerosis and their first-degree relatives: a nationwide cohort study in Denmark. Mult Scler.

[102] Kimura K, Hunter SF, Thollander MS, Loftus EV, Melton LJ, O'Brien PC (2000). Concurrence of inflammatory bowel disease and multiple sclerosis. Mayo Clin Proc.

[103] Gupta G, Gelfand JM, Lewis JD (2005). Increased risk for demyelinating diseases in patients with inflammatory bowel disease. Gastroenterology.

[104] Singh S, Kumar N, Loftus EV, Kane SV (2013). Neurologic complications in patients with inflammatory bowel disease: increasing relevance in the era of biologics. Inflamm Bowel Dis.

[105] Stovicek J, Liskova P, Lisy J, Hlava S, Keil R (2014). Crohn's disease: is there a place for neurologic screening?. Scand J Gastroenterol.

[106] Ramagopalan SV, Dyment DA, Valdar W, Herrera BM, Criscuoli M, Yee IM (2007). Autoimmune disease in families with multiple sclerosis: a population-based study. Lancet Neurol.

[107] Surtees R, Clelland J, Hann I (1998). Demyelination and single-carbon transfer pathway metabolites during the treatment of acute lymphoblastic leukemia: CSF studies. J Clin Oncol.

[108] Ding T, Ledingham J, Luqmani R, Westlake S, Hyrich K, Lunt M (2010). BSR and BHPR rheumatoid arthritis guidelines on safety of anti-TNF therapies. Rheumatology.

